# On the outside looking in: a phenomenological study of the lived experience of Australian adults with a disorder of the corpus callosum

**DOI:** 10.1186/s13023-021-02140-5

**Published:** 2021-12-14

**Authors:** Maree Maxfield, Monica S. Cooper, Anne Kavanagh, Alexandra Devine, Liz Gill Atkinson

**Affiliations:** 1 Centre for Health Equity, Melbourne School Population and Global Health, The University of Melbourne, Level 4, 207 Bouverie St, Melbourne, VIC 3010 Australia; 2 Neurodevelopment and Disability, Royal Children’s Hospital, 50 Flemington Rd, Parkville, 3052 Australia; 3 Melbourne Disability Institute, Melbourne School Population and Global Health, The University of Melbourne, Level 4, 207 Bouverie St, Melbourne, VIC 3010 Australia; 4 Disability and Health Unit|Centre for Health Equity, Melbourne School of Population and Global Health, The University of Melbourne, Level 4, 207 Bouverie St, Melbourne, VIC 3010 Australia

**Keywords:** Corpus callosum, Rare disease, Lived experience, Phenomenology, Disability, Agenesis of the corpus callosum, Adults, Heterogeneity, Hidden disability

## Abstract

**Background:**

While classified as a rare condition, a congenital disorder of the corpus callosum (DCC) is one of the most commonly identified brain anomalies in newborns, occurring in 1:4000 live births. Advances in imaging techniques have improved early diagnosis for children, yet adults with a DCC—who may present with extreme heterogeneity in cause and impact—often experience challenges in receiving a definitive diagnosis and accessing appropriate services and supports. To date, the dearth of evidence documenting the lived experiences of adults with DCC has made it difficult to determine adequate policy and service responses. This exploratory research aims to address this gap by presenting the first qualitative examination of the experiences and impact of complete or partial agenesis of the corpus callosum among adults.

**Results:**

Eight face-to-face interviews were conducted with Australian adults, aged 23–72 years, to explore their lived experience. Data was collected in four Australian states from June to August 2017. Thematic and interpretive analyses were employed to analyse data. Three emergent themes described difficulties related to: (1) reactions to the diagnosis; (2) access to supports and key life domains, and (3) identifying as an adult. Interview analysis described lived experiences typically outlining a lifetime of exclusion and misunderstanding from family, educators and disability and health support services.

**Conclusions:**

This paper contributes to filling the knowledge gap around a rare congenital brain disorder affecting the lives of adults. Findings confirm a considerable lack of information and support for adults living with corpus callosum disorders. Greater professional and societal understanding is needed to improve access to the key life domains of education, employment and social inclusion for adults with a DCC. To instigate truly effective change, social research must tackle the issues of applicability and impact to alter the dominance of uninformed practices, hindered by prevailing myths. This research paves the way for further phenomenological studies in which participant narrative is vital. Further research will elicit stronger policy and service responses for all current and emerging adults with a DCC.

## Introduction

Disorders of the corpus callosum (DCCs) are one of the most commonly diagnosed congenital brain disorders in newborns, yet they remain poorly understood within health systems and the wider community [14]. Even less is known of how congenital DCCs affect the lives of adults. With an incidence of approximately 1:4000 live births, a DCC is classified as a rare disease [16, 20]. There are no data informing prevalence of adults diagnosed in adulthood. DCCs are heterogeneous in cause and presentation [14] and are inconsistently described and conceptualised [23].

DCCs affect cognitive, physical and psychological functions with impacts ranging from mild to severe [29, 43]. Current paediatric management practices are informed by clinical and behavioural research [5, 12, 44]. However, there is limited research to inform management and support practices for adults living with a DCC. Indeed, there is a paucity of information regarding their lived experience in relation to support needs and whether these are being adequately provided by professionals, families and the wider community.

This study is unique, being the first to qualitatively examine the lived experience of a sample from the population of adults with a DCC. The purpose of this research was therefore to explore how congenital DCCs affect the lives of Australian adults diagnosed with the condition. Our paper begins with an overview of disorders of corpus callosum, summarizing key presentations. Following a description of the research methods, we present findings from our study, drawn from eight individual face-to-face interviews with Australian adults with a DCC.

## Background

### What are disorders of the corpus callosum?

The corpus callosum is the largest white matter structure connecting the two hemispheres of the brain [43]. It enables cognitive, motor and sensory messages to be transferred between the hemispheres [5, 35]. A DCC is diagnosed by ultrasound, magnetic resonance imaging (MRI) or computed topographic (CT) brain scan.

This study uses the collective term, disorders of the corpus callosum or DCCs. The term, DCC, could be considered the broad umbrella term to include agenesis of the corpus callosum (ACC) (which technically means a completely absent corpus callosum), partial agenesis (partially absent corpus callosum), dysgenesis (misshapen corpus callosum) and hypoplasia (thin corpus callosum) [14]. Consistent nomenclature has not yet been established in the medical, research or DCC communities and hence the terms ACC and DCC are used interchangeably.

Historically, knowledge of DCCs relied on clinical studies with small numbers of people. Some medical professionals maintained that individuals with a DCC were typically asymptomatic, developing normally and without developmental delays [49]. Others disagreed, suggesting DCCs are associated with a range of clinical characteristics. In a parent reported study of 678 individuals aged from 4 months to 45 years [41], Schilmoeller and Schilmoeller noted the presence of one or more cognitive, physical or psychological functional difficulties in each individual. The difficulties included affected language, communication, processing information, learning, vision, muscle tone, eating and elimination. Associated conditions reported included cerebral palsy, seizures, microcephaly, Obsessive Compulsive Disorder (OCD) and Attention Deficit Hyperactivity Disorder (ADHD). Additionally, Autism Spectrum Disorder (ASD) or autistic-like behaviours were identified, for example, difficulties with change and abstract reasoning. This study also highlighted that although children were described as ‘happy,’ only 22 per cent enjoyed interacting with their age peers. Very few parents (10%) reported being satisfied with the knowledge, clinical treatment and support availed by medical professionals [41]. Subsequent studies have supported these findings, documenting cognitive and psychosocial impairments [1, 6, 38, 50], problems with learning and memory [15, 37] and neurodevelopmental delays [5, 29]. Other researchers have also described comorbidities with DCC such as ASD, epilepsy and cerebral palsy [25, 42].

A systematic review of 47 peer reviewed articles, from 1980 to 2011 [43], aimed to build a neuropsychological profile of individuals with DCC. The authors concluded that individuals with full or partial agenesis of the corpus callosum typically had intellectual functioning below that of the general population and experienced a wide range of neuropsychological impairments with risk of disability. Finally, in a publication describing the neuropsychological syndrome of corpus callosum disorders, noted researchers, Brown and Paul [5], identified three key deficits: (1) A reduction in cognitive processing speed, (2) Slower transference of sensory motor information between the brain’s hemispheres, and (3) Reduced capacity for complex reasoning and novel problem solving. In adults this presents as a spectrum reaching the areas of intellectual disability, anxiety and poor social skill acquisition.

### Understanding rare disease and disability

Research suggests that people with disability often experience poorer social and economic outcomes when compared to people without disability [13, 21, 22]. Similarly, studies of children and adults with rare diseases demonstrate that reduced quality of life and social disadvantage are common [4, 51]. Congruent with other rare diseases*,* disability associated with a DCC can impact the socio-economic and quality of life of individuals [2, 33].

Individuals with a DCC are at risk of experiencing chronic socioeconomic disadvantage, affecting health and wellbeing and reducing the capacity to attain full potential [33, 51]. Yet, as with other rare disease, there is a paucity of empirical evidence. This makes it challenging for health and educational professionals, systems and resources to appropriately support people with DCCs. Knowledgeable, empathetic and tolerant professionals with access to evidence-based information are indicated as significant factors in reducing stress and providing effective management [33]. These factors are currently lacking in the Australian context.

### The importance of understanding lived experience

Lived experience allows interpretation and understanding of the lives, choices and social world of others [19]. It enables understanding through ‘experiential concreteness, vividness and descriptive detail’ ([48]: 810). Adults with DCCs have not had the opportunity to collectively communicate their lived experience. Clinical research describes complex impacts of DCCs. However, that does not translate to how that complexity affects the lives of adults with DCCs from their perspective. Views and needs can differ significantly between people with disability and those who support them. Additionally, they can also differ within the disability community [46]. Australia is a signatory to the UN Declaration [46] for people with disability to have equal access to choices, support and participation in their local communities. To exercise that right, it is important that their voices are heard. Flynn [18] states that ‘hearing one another’s personal experience promotes an atmosphere of cooperation, deep listening and solidarity’ (p. 15), providing opportunities for research to explore the complexities of DCC impacts.

The voices of adults with a DCC have been under-represented in research to date. There is little evidence of the impact of DCCs on the lives of Australian, or any, adults with a DCC and whether they are appropriately supported to reach their full potential across key life domains. To our knowledge, this is the first study which aims to understand these experiences from the perspectives of Australian adults with DCCs.

## Research process

To ensure an ethically respectful interpretation of their narratives, we acknowledged the historical marginalisation when considering an appropriate research methodology. A phenomenological approach was selected to ‘understand, describe and interpret human behaviours and meanings individuals make of their experiences, communicated in their own terms’ ([8]: 117). Phenomenology offers an effective qualitative methodology to assist marginalized and minority groups to be heard [9, 11, 24]. It allowed this study to elicit rich, in-depth data to address the research question: ‘*What is the lived experience of Australian adults with a disorder of the corpus callosum?*’ through collecting first-hand narratives.

Ethics approval (1748572.1) was granted by the Human Research Ethics Committee, School of Population and Global Health at the University of Melbourne. Written or verbal informed consent was obtained from all participants with the option of an accessible, ‘Easy English’ version.

### Sample, recruitment and data collection

Adults with DCC were recruited using purposive sampling [19]. Participants were identified and selected from responses to advertisements in relevant support groups and through neurology departments. Individuals were included if they were 18 years or older, had a DCC diagnosed with neuroimaging, able to give informed consent and to participate in an interview of approximately one hour. Given the limited scope, resources and time frame of the project, we limited the sample to eight eligible respondents. They represented diversity across diagnosis, age, gender, geographic location, relationship status, occupation, socioeconomic status and educational levels [11, 27].

A total of eight participants five female and three males aged 23–72 years (average age 44) participated in this study. All participants had received a diagnosis of a DCC as adults (18 or older) apart from one, who was 16 at the time. Reported diagnoses included complete agenesis (n = 2), partial agenesis (n = 4), dysgenesis (n = 1) and hypoplasia (n = 1) of the corpus callosum (Table 1). The participants were residing in urban and regional areas across four Australian states. Semi-structured, face-to-face interviews explored health and wellbeing, education, employment, interests, relationships, social inclusion and advice to others. Interviews ranged in length from 47 to 84 min.

**Table 1:** Summary of participant demographics and reactions to diagnosis

Pseudonym	Education (completed)	Current paid employment	Diagnosis	Age at dx (Yrs)	Doctor reaction	Participant reaction
Leigh	Yr 11 (MS)	No	Dysgenesis	53	Dismissive. No knowledge. Doctor Googled in apt. Advised Leigh to keep silent about it. No information	Enraged, angry, frustrated
Robin	Yr 12 (SES)	No	pACC	16	Advised parents that Robin couldn’t drive. No other information available	Shocked, Surprised, annoyed
Claire	UG Deg	Yes	ACC	18	Doctor notified parents who told Claire. Ignored	Surprised, unsupported
Ash	Yr 10 (MS)	No	pACC	20	Notified of a birth defect. No prior experience among treating doctors. No other information	Shocked, confused
Shannon	UG Deg	Yes	pACC	19	Never seen it before. Dr Googled in apt. Told Shannon not to worry and nothing could be done about it	Detached, worried about possibly related issues
Simon	UG Deg	Yes	pACC	33	No information available. Talk to GP	Vindication, an explanation, isolated
Kim	Yr 11 (MS)	No	ACC	40	No information given	Shocked, alone, relief. Always knew something was wrong
David	UG Deg	No	Hypogenesis	61	Plans to monitor corpus callosum changes. No other information	Curious and interested

Apt, appointment; MS, mainstream schooling; SES, special education setting; dx, diagnosis; pACC, partial agenesis of the corpus callosum; ACC, agenesis of the corpus callosum; UG, undergraduate; Yr, year

### Data analysis

To augment description with interpretation, two phenomenological approaches were employed for data analysis. The first was thematic analysis. A coding framework for thematic analysis allowed the dissection and reduction of transcribed, experiential data through exploration and integration of codes, to identify themes [39]. The second, Interpretive Phenomenological Analysis (IPA), assists the researcher’s capacity to understand an individual’s lived experience through detailed examination. It was interpretive because of the additional recognition of the researcher’s role of interpreting the participant’s narratives while identifying meaningful experiences in the individual’s personal and social world [11, 45].

It was important to consider ‘insider’ and ‘outsider’ positions to maintain impartiality through balancing researcher subjectivity and objectivity. Introduced by Merton [32], the concept of ‘insider’ and ‘outsider’ status in research describes the degree to which a researcher is positioned either within or outside the community being researched. Insiders are regarded as having greater connection and insight to the group they are examining, whereas outsiders enter the research with no prior connection. It is believed that while an outsider may offer a more objective evaluation, the subjectivity of an insider may promote greater understanding.

As the parent of an adult with a DCC and considered an ‘insider,’ the lead researcher considered bracketing, a process of suspending all prior knowledge and assumptions. This process aims to alleviate insider bias and promote objectivity for researchers [8, 47]. Barriers personally encountered as a caregiver, underpinned by lack of recognition and support for adults with a DCC, provided the initial motivation for this research. Denzin and Lincoln [11] remind us that subjectivity can become entangled when assessing other people’s lives. Nevertheless, as social researchers, we are part of the social world we study [17]. Within this research, a degree of subjectivity was regarded as beneficial for ‘insider’ awareness, assisting with identification of under-recognised themes and situations in this community. The four co-authors involved in design, analysis and reporting were ‘outsiders,’ although one has a disability that is not a DCC.

To maintain rigour and assist with directing the impact of the researcher’s preconceptions and personal feelings, reflexivity was instrumental during design, data collection and analysis. Mauthner and Doucet [31] reason that data analysis and interpretation techniques are not separate, neutral techniques. They are a reflexive exercise through which meaning must be made rather than found. Reflexive strategies employed by the lead researcher included engaging in discussions with ‘outsider’ supervisors and research colleagues, systematically and critically maintaining a reflective journal and seeking advice from family support organisations.

Data analysis was conducted using thematic analysis methods informed by Saldaña [39] and Carpenter [8]. Interviews were transcribed verbatim. Pseudonyms were substituted during transcription and demographic data recorded, with specific identifying data minimised to preserve privacy and identity. Analysis was conducted by reading and re-reading both digital and print formats, employing highlighted colour coding to easily identify broad themes, sub themes and codes. Colour coded ‘Post-It’ notes were used to visually sort and resort.

**Fig. 1:**
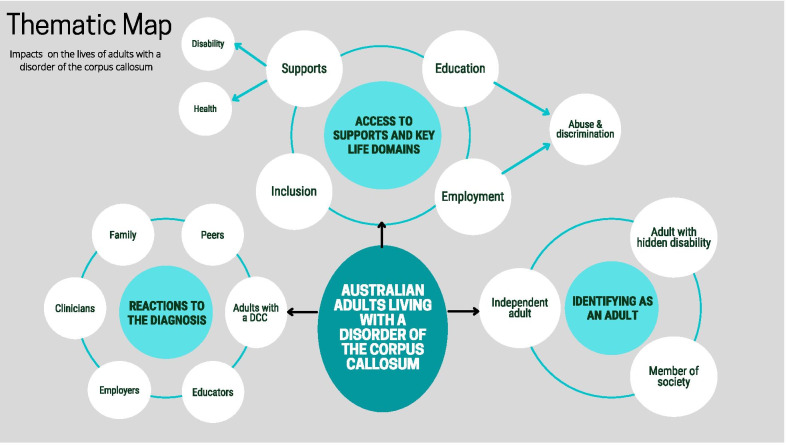
Thematic map

The information was digitally tabulated as a coding framework using Excel and visually represented as a thematic map (Fig. 1). Codes were reviewed individually and sorted within sub-themes to enable an explanation of the meaning of the data and how they related to three key themes (For example, being diagnosed with a DCC prompted reactions from a broad range of people in participants’ lives, generating the first key theme, ‘Reactions to the diagnosis.’ Analysis revealed that most participants were shocked or angry to learn of their DCC diagnosis, whereas other people’s reactions were mixed and often indifferent and unsupportive. Examination indicated that reactions of a range of other individuals, including clinicians, family, friends and employers, were described by all participants with examples.

Rich data enabled in depth exploration of the key themes. Data analysis involved authors, MM, AD and EGA and consensus was reached through discussion, with no disputes arising. The process identified three key themes; (1) Reactions to the diagnosis, (2) Access to supports and key life domains, and (3) Identifying as an adult (see Fig. 1).

## Results

Analysis revealed that no participant had received a DCC diagnosis in childhood. Participants were aged between 16 and 61 years when they received their diagnosis. All had undergone MRI to explore incidental clinical presentations including seizures, injury, chronic headaches, anxiety and autism. Reporting of their diagnosis by medical professionals was generally regarded as insensitive and unsatisfactory. Being told that they had a hidden ‘birth defect’ in their brain, which was subsequently trivialized or dismissed, brought a range of responses that increased their uncertainty and anxiety about the condition.

### Theme 1: Reactions to the diagnosis

The first theme centred on the reactions of participants and others, to the confirmed DCC diagnosis. For participants who had experienced unexplained lifelong challenges, the diagnosis marked a significant point in their lives. They were optimistic that such an irrefutable diagnosis would elicit compassion from others, enabling improved access to support and understanding. To the contrary, many encountered judgmental attitudes, indifference and hostility. They described the challenges of having to process their own reactions in addition to the unexpected reactions from family, peers, colleagues, educators and employers.

All participants were unaware of their DCC prior to incidental, investigative neuroimaging. It was not intentional to focus on people with DCC who were diagnosed in adulthood and this presented an unexpected and interesting finding. The discovery that a significant part of their brain had been missing since birth prompted a variety of reactions including surprise, confusion, dismay, anger and shock:

Yeah, when they said that this part of my brain was missing, I thought, ‘What? What part of my brain’s missing?’ You know? I just had that sort of bobbing in my head. It was just crazy. I felt shocked that I didn’t have part of my brain there and not knowing that I didn’t know that part of my brain wasn’t there (Robin).

I was enraged that in 53 years nobody had told me something that’s an explanation for so many things in life since birth… I was angry and I was (long pause) I was sort of taken aback and thought, ‘Does it mean I’m mad? Does it affect your cognitive abilities? Does it affect your intelligence? Does it mean I have low IQ? Would life have been different without it or would life have been different had I known I had it? (Leigh)

Two participants described feeling relieved and validated after discovering a reason for lifelong, unexplained social, cognitive and physical difficulties, expressed by Simon as, ‘It shows how much I’ve had to overcome.’ Although six participants expressed a strong belief that the DCC had caused the difficulties, one participant identified associated traits as possibly connected but more likely to be coincidental:

Well, I consulted Dr Google … the symptoms appeared to be from nothing, almost nothing, which is me, to people who had very severe effects. Looking back, well of course you can ascribe all sorts of things to it. There’s a range of things there but not enough to draw any strong conclusions (David).

All participants reported that the diagnosing clinicians admitted that they had no prior knowledge or experience of DCCs. Clinicians consulted search engines during the consultation or advised participants to go home and do their own internet research. Five participants reported being told to ignore the diagnosis as it wouldn’t affect them or there was nothing that could be done about it. All participants expressed a degree of dissatisfaction with the delivery of their diagnosis due to insensitivity and a lack of knowledge by the clinicians. They reported being offered no information (n = 6) or misinformation (n = 2):

He told me that I've got this thing. He said, “I don't know what it means.” … He turned his screen around so we could see what came up on Google … It was sort of like, don't worry about it because there's nothing we can do about it anyway but I did worry about it because I had other diagnoses at the time. I wondered if that was somehow related (Shannon).

Although surprised by the lack of professional understanding, participants expected family support. Shannon described support from a sister:

I have a sister and we were always pretty close. I think she was pretty supportive of me as a kid and later as well. She’s not changed since the diagnosis. I think she might have a bit more understanding but I think my whole family didn't really know what to do with the information.

Conversely, more than half the participants expressed disappointment in family members’ reactions. They reported that some family members used the diagnosis to humiliate, exploit or dismiss them:

It [DCC] was always thrown in my face when there were issues. It was always thrown in my face and I was put down. People talked behind my back. I knew it and felt embarrassed by it. I felt really upset about it for a long time. I couldn’t talk about it (Ash) .

Mum made me Power of Attorney. Anyway, my sister wanted to take over from me and have me declared ‘unfit.’ She talked to my doctor and my doctor said to me afterwards, “Don’t ever let her know you’ve got this ‘thing’ because she’ll use it against you” (Leigh).

Mum just said, “It’s bullshit. Just forget about it,” because what you didn't understand you just put aside and didn’t think about (Leigh).

Participants expressed difficulties in deciding whether to share their diagnosis and with whom. Some regretted entrusting a friend or employer with details of their diagnosis. For example, Leigh described people’s reactions when sharing the diagnosis as ‘just glazing over.’ Participants repeatedly described feeling patronised, dismissed or inadequate and believed that people’s comments invalidated the diagnosis, as summarised by Claire, ‘Everybody I meet, they all say, “But you’re normal, you’re bubbly, you’re outgoing*”’.*

Participants were re-evaluating their lives. They wondered how a DCC had affected their opportunities for social inclusion, quality education, meaningful employment and successful personal relationships. These impacts are explored in the second theme.

### Theme 2: Access to supports and key life domains

The second theme relates to findings associated with access to key life domains. Chronic failure to access domains of education, employment, physical and mental health and social inclusion was evident. The range of issues and obstacles encountered illustrated the heterogeneity of DCCs. Participants reported a range of associated comorbidities and deficits which were identified as impacts of DCC requiring support. They expected that receiving an irrefutable diagnosis would create greater access to supports and inclusion.

#### Access to supports and inclusion

Nevertheless, most participants reported frustration and chronic failure when trying to access systemic health, disability, educational and financial supports. Negotiations were described as arduous, stressful and futile. Seven participants described mental health issues which included anxiety, depression and/or suicidal ideation:

I am diagnosed with anxiety, depression, suicidal … I’m not coping well. I’ve fallen down the hill. The council have handballed me to the NDIS [National Disability Insurance Scheme]. The NDIS have handballed me back. There’s a group that are trying to advocate for me but they're all about just getting money from the NDIS and handling the money (Leigh).

I had a nervous breakdown. I was assigned some caseworkers because of my situation, I had to go through the children's court through those two years. They thought that I'd never have my kids back (Kim).

David indicated that it had little effect and supports and management were not required, ‘It hasn't made any difference to the sorts of things I’m able to do or the perceptions I've had or the things I enjoy.’ To the contrary, the other seven participants expressed the need for effective, ongoing, practical and social support:

I'd like a caseworker. Somebody who can check on me from time to time just making sure that I'm doing what I'm supposed to be doing. I would say it's pointless applying for the NDIS (Kim).

I would love to have it [support]. Yes. Even if someone could come here and either drag me to the place or there’s someone that comes to the home. I’ve got no professional support. I’ve got my daughter’s support and that’s it! (Ash)

Barriers to support included the paucity of research, participants’ self-professed lack of social sophistication and professionals’ insufficient knowledge of DCCs. Leigh indicated that not one of her health professionals had ever heard of DCC. She described feeling like she was drowning and had no direction:

It’s like being given a magic box but there’s a trick to opening it and you don’t have the trick but it does affect me and I feel like I’m in a forest and I can’t see anything, just darkness and trees. I don’t even have a direction to go in with it.

Misunderstanding social cues and not being able to keep up with peers were reported by six participants. They recalled difficulties making and keeping friends. Sensory issues and miscommunication were mentioned as social inhibitors, with almost all participants (n = 7) reporting problems with anxiety. All participants provided examples of social isolation, teasing or bullying during their school years and felt that this had generally continued into adulthood:

I wasn’t socialising with the other kids. I was choosing to play in the playground by myself (Shannon).

I’ve been bullied all my life, being bullied again and again and not fitting in. Being bullied by some of the lecturers and some of the students, you know, just being completely isolated (Claire).

Family relationships, both positive and negative, played a key role in the lives of all participants. Participants referred to physically and sexually abusive relationships with family members. Some were excluded by family members.

I was always the kid that was a bit on the outer, never fully part of the group. You're a target for bullying, although the worst bullying was from one of my relatives (David).

Conversely, others felt that their family members actively assisted them to seek social opportunities and provided much of their companionship. Three participants identified one person in their family who provided ongoing support and assistance. More than half the participants reiterated that they constantly felt alone, didn’t belong and did not have close friends. Mothers were mentioned frequently. A few were described as supportive. For example, Simon described support in gaining employment, ‘Getting a job? Again, Mum knew someone who was doing it and they sort of got talking one day.’ Conversely, participants offered instances of mothers compounding issues, for example:

I've always got Mum in the back of my head. She's always there telling me not to do this and not to do that (Claire).

My adoptive mum kicked me out of home because I was corresponding with my birth mum and she didn’t like it. I wasn’t the perfect child so she would scrap me and focus on my sister (Kim).

Belonging to a community had presented some long term obstacles. Key barriers included difficult family relationships, being regarded as ‘different’ or ‘weird’ and not understanding social expectations. Not being accepted or ridiculed for being different were exemplified by Claire:

I was never invited to birthday parties and things. That’s why I never truly felt part of the group. I always felt that I'm on the outside looking in as opposed to truly being included … a little bit of bullying in the playground, like kids pulling up my dress and pulling down my undies sort of thing. A little bit of, ‘Oh, she’s strange,’ and whatever. That was horrible (Claire).

Participants believed that the impacts of DCC had affected their capacity to gain employment and adequately support themselves. More than half relied on a government Disability Support Payment (DSP), regarding it as inadequate to establish an acceptable quality of life, raise a family or access local and wider communities. Some had been hopeful about the implementation of the National Disability Insurance Scheme (NDIS), which had been introduced by the Australian government in 2013, responding to the needs of people with permanent physical or psychosocial disability. The NDIS supported individuals requiring specialised care, with emphasis on community engagement, education and a happy daily life. Two participants had applied for support but had been rejected. Another who had been successful felt pressured to accept minimal supports that did not appropriately meet their DCC needs. Others had given up because they had insufficient documentation to make an application. Although NDIS focuses on functional impacts, gaining access includes stating primary disabilities which are matched to lists. DCCs are unrecognised by the scheme and do not appear on any lists. Difficulties with self-advocacy were illustrated by Shannon:

My problem is that I don’t know what support I need. I don’t know what help I need. That is the problem right there. I don’t know how to sell my point of view in a way that makes it connect.

#### Access to education

At the time of diagnosis most participants had completed formal education. More than half the participants had repeated at least one year level. They believed their education experiences influenced their current position with some reporting feeling inadequate and inferior. They described problems with communication, anxiety, isolation and negative reinforcement as key educational obstacles. Low expectations from educators and family members had also hampered their academic achievements. For example:

The teacher said to Mum and Dad, “There’s no use this child even doing maths. She’s mathematically illiterate. She’ll never learn a thing” (Leigh).

Someone could explain something to me until they’re blue in the face. Mum got frustrated at telling me what I had to do over and over and over and over again. I’d study hard and try really hard but nothing would sink in and I knew something was not quite right (Kim).

In contrast, others described support from parents and teachers to pursue goals. Half the participants had completed a university degree and expressed the belief that they had achieved academic success through developing strategies of perseverance and resilience to overcome obstacles. Claire reported being set up for failure:

Everybody was trying to build me up for failing and I didn’t want to fail. I wanted to pass. I wanted that bit of paper.

Although Claire persevered and achieved a university entry score, she felt that ensuing ‘congratulations’ were patronising and laced with incredulity and disbelief:

The school counsellor involved was my next door neighbour … She was amazed and shocked and still is, at how well I’ve done. They are meaning well but it’s like, she doesn’t know the half of it! It was horrible! I remember describing it like being a piece of string being pulled in every imaginable direction at once and through the dirt (Claire)

#### Access to employment

Learning difficulties and poor educational attainment affected participants’ capacity to gain meaningful employment. Three participants were engaged in paid employment and felt that their determination to complete educational goals had enhanced their employability. David had a long career employed in a senior position. However, he described difficulties with interpersonal engagement at school and in the workplace:

One of the issues is that they [adults with DCCs] have difficulty with finding employment and if they do find it, they have trouble maintaining it …. One of my areas of weakness was probably interpersonal skills and the higher you go the more important they, apparently, are… I was shaped by my experiences at school. I had difficulty getting on with other kids.

Five participants had either ceased or never participated in paid employment, citing lack of skills, anxiety, poor educational outcomes and lack of supported opportunities. Ash described being actively discouraged by employment agency staff:

I said, ‘Look, I need a job. I need you to help me get into a job.’ She was looking at me, she was looking at all my work, my scan and everything and she said, “You know, you don't have to work for a day for the rest of your life”.

Participants believed that an earlier diagnosis and better educational outcomes would have led to greater employment opportunities. Those who had been employed expressed some difficulties keeping up with demands of their jobs. Two participants indicated that disclosure of their DCC had worsened the situation with unwarranted consequences including negative attitudes, demotion and bullying. Claire described bullying by both employers and colleagues:

I've been bullied all my life. I try and talk to bosses about things and particularly about my brain and I'm almost in tears and trying to keep the lid on it. I had issues with employment. In fact, that whole period is all kind of traumatic for me, the way I was handled and treated and everything … It’s such a toxic environment that I’m in (Claire).

Ongoing obstruction to accessing key life domains leads to the third theme, which describes the impact on participants’ identities as adults.

### Theme 3: Identifying as an adult

The third theme explores findings related to identity. Identity refers to the self and the expression of individuality as one navigates through the tasks of daily living [26]. Participants self-identified as independent adults, people with a hidden disability and members of society.

One participant felt that a DCC had minimal impact on their identity as an adult. In contrast, six participants found adulthood challenging and expressed feeling immature and facing ongoing difficulties fulfilling societal expectations. They described their struggles to be adults:

I don’t know how to ‘adult.’ I’m having a really hard time. It’s the ACC, 100%. I’m feeling really sick about it. I feel like I have to do it because I am the adult of the household (Ash).

I see myself as being quite immature when I started uni. I knew that I was… I think socially, I was also immature and probably am now and probably always will be (Shannon).

Although some participants were parents (n = 3), in relationships (n = 3) and/or had paid employment (n = 3), they expressed difficulties with demands of adulthood. They felt they were not adequately equipped to meet the responsibilities and societal expectations of independent living. Problems with organising finances, household management, maintaining relationships and raising children were exemplified. More than half stated that they were confident with managing finances. Others experienced problems and had family members helping them:

Just the one thing that I am finding really hard right now, being an adult with ACC, is the coping with the bills and putting everything together. Sometimes I get my daughters to help me out and they go, “Yeah but you gotta do it. You can’t neglect it.” I actually did neglect all my bills at one stage (Ash).

Restricted mobility featured as a barrier, undermining capacity to be an independent adult. The majority of participants could not drive. Some had failed repeated attempts to secure a licence. The three who held a driver’s licence had all been involved in collisions and took extra precautions such as driving at quieter times. Not driving affected access to services and exacerbated social isolation, particularly for single parents. Participants outlined obstacles to adult responsibilities such as shopping, employment, social activities and transporting children:

We were out the door at 7:30. And then I'd start. I'd walk. I'd walk all the way up [street] to the school. It's about eight ks [km] there and I'd walk that twice a day, well, four times. There, back, there, back and I remember that hill with a stroller, a double stroller with, you know, [child] in those little, um, pouch things, going up that hill (Kim).

Some participants found sustaining adult relationships with partners and family members problematic. Although they had developed resilience, they needed support that wasn’t readily available. The majority of participants described ongoing anxiety, depression and experience of episodic mental illness and/or suicidal ideation. Although two participants felt they were in stable relationships, seven indicated that they would like more support. All participants reported degrees of bullying and/or abuse at school, in the workplace or in the home. Bullying had destroyed confidence and self-worth. Abuse was verbal, emotional, physical and sexual. For some, this abuse had ceased after childhood but for others it continued into adulthood. Kim shared her experiences:

He was an abuser. Beat up, you name it, abused the kids, abused me. Yeah, we’re talking nasty. Broken bones…

Participants mentioned oscillating between feeling ‘normal’ and ‘not normal.’ They were required to function and conform to socially constructed expectations but were ostracised because of differences which had no visible cause or reason. Ash explained the confusion of hidden disability. ‘If my brain was on the outside you still couldn’t tell because you have to split the brain in the middle to see that, don’t you?’ Claire described the difficulty of operating in the two realms as, ‘My soul’s been laid bare and then I've got to just flip a switch and I'm ‘normal’ again.’

Participants embraced their rare diagnosis, describing how a diagnosis finally offered reasons for behaviours and impacts. They recognised differences in themselves which were not readily understood by others. Some expressed resentment at the years of mismanagement and the lack of control of ‘ownership’ of their lives because others had dictated how they should factor any deficits into their identity. Although they were grappling with its meaning, they regarded their diagnosis as an important part of their identity:

If all of my problems are due to ACC and it's this physical thing that I can't change, it's like part of who you are as an individual, this problem that you've got to solve ... It makes me an individual but I see those problems as problems that need to be solved. There’s a dichotomy between the two because those problems are probably what it means for me to have ACC and that makes me who I am. I'm having trouble making the two coexist (Shannon).

All the participants indicated that DCCs needed greater recognition, acknowledgement and support. Two participants spoke about discovering the fledgling corpus callosum support group, Australian Disorders of the Corpus Callosum (AusDoCC) and hoped it would grow to offer greater support and connection. Support groups are recognised as valuable for information, building advocacy and in creating a community for people with rare conditions [2, 34]. Although all participants spoke of developing resilience and coping strategies, they identified many obstacles. Living with an invisible, under-recognised and largely unsupported condition had affected their inclusion and identity:

Yeah. As Mum said, “You're a trailblazer. You're a pioneer.” I'm sick of being a pioneer! It's really stressful. Listen to us because we all are affected. There are some commonalities but we are also affected in individual different ways. Listen to us (Claire).

## Discussion

Congruent with experiences of people with disability and rare diseases more broadly, the participants with corpus callosum disorders typically encountered delays in diagnosis, misdiagnosis, poor recognition and inadequate support [2, 7, 10, 33, 51]. Additionally, adults in this study described further similarities to people with disability and rare diseases through encountering barriers to important requirements for meaningful lives—education, employment, relationships and societal inclusion [4, 13, 21, 22].

These findings contribute important data to a knowledge gap related to Australian adults with a DCC. They add insight to how being diagnosed with a corpus callosum disorder has affected their lives. Their narratives indicated that there were issues during childhood that retrospectively demonstrated unrecognised and unresolved impacts of a hidden disability. Many of the impacts were apparent in childhood but no accurate diagnosis was made. Upon reflection, they were indicative of a corpus callosum disorder but the knowledge and sophisticated imaging techniques were not readily available to provide an accurate diagnosis.

Unlike some neurological conditions, the eventual diagnosis of a DCC for the adults in this study, confirmed by neuroimaging, was indisputable. However, the rarity and heterogeneity of DCCs, in cause and presentation, meant that professional knowledge was fragmented and contradictory. Prognosis was difficult and individuals and families were given inadequately informed advice. There is still no empirical evidence to support the misconception that was typically proffered by clinicians that there are “thousands of perfectly normal people walking around with no corpus callosum” ([40], 225). Scientific literature describes a range of deficits with DCCs which concurred with participants’ descriptions of impacts and impairments.

After receiving the diagnosis, adults in our study expressed frustration at continued invalidation or dismissal by professionals, family members and others. Although the diagnosis had provided an explanation for lifelong problems, validation was not typically reflected in the reactions of others. Many earlier key life decisions were made, based on inaccurate observations and uninformed scientific evidence. They failed to correctly acknowledge challenges and impairments. Participants were told that nothing could be done about that and to continue on with their lives. However, it was a pivotal life moment which validated their personal struggles and prompted a major re-evaluation of their lives. Yet, they remained unsupported. Problems of abuse, unemployment, poor mental and physical health and social isolation prevailed.

Participants reflected on the implications before and after diagnosis. A diagnosis offered some explanations for their lived experiences. Evidence indicates that DCCs affect key childhood domains, particularly learning, developing friendships, developing physical skills and belonging [3, 44]. Two key factors in the effective support of individuals with rare and chronic conditions are accurate information and effective professional management [2, 51]. It was apparent that throughout childhood and adolescence the participants had neither. Some experienced a sense of loss from problematic childhoods without having valid explanations for their ‘differences.’ They had not been protected from abuse at school, in the family home and in workplaces.

Our study demonstrates barriers to accessing a range of social supports. Three participants mentioned trying to unsuccessfully access the NDIS which was incrementally being rolled out across Australia at the time of interviews. Some felt that the NDIS may have provided the change they had been waiting for but little was known about it. People with more readily recognised disabilities and documented histories appeared to gain easier access to the scheme. Those who had been unsuccessful felt rejected and misunderstood.

Some participants described experiences of repeated failure and obstacles to educational opportunities, feeling unsupported and actively discouraged. Bullying and social exclusion were also prevalent. Corpus callosum literature specifies deficits in complex reasoning skills, slower brain interhemispheric transfer and reduced cognitive processing [5]. Learning difficulties described, commensurate with evidence for DCCs, included production lags, communication and problem solving [6, 37, 44, 50]. Examples of these deficits were reiterated in participants’ narratives. Educational impacts were also apparent through the high incidence of repeated year levels indicating a realm requiring further investigation. It was demonstrated that reduced capacity for interhemispheric communication had affected cognitive, behavioural and social functioning. Neuropsychological testing was not mentioned by participants but may have been beneficial to assist educational and other supports through identification of deficits and strengths. Recapitulating the impact of a DCC, Paul [36] states, ‘If you don’t have a corpus callosum, you’re not going to be able to have information go back and forth, between the hemispheres, as effectively as someone who was born with a corpus callosum.’

Academic outcomes affect employment opportunities and the capacity to earn income. Participants reported that inadequate educational opportunities had reduced their capacity to access well-paid employment, creating financial burdens. These were aggravated by cognitive delays and the psychosocial impacts of DCC. For some individuals, childhood bullying and social exclusion continued into the workplace. Access to education, employment and social inclusion are key social determinants, vital for health and wellbeing [30]. The impacts described by participants impeded their access to key life domains and functionality as independent adults, particularly those who were parents.

Anxiety experienced as children leads to other mental illness such as depression and suicidal ideation in adults [7, 28]. This was evident in our study cohort. Lifelong supports were inconsistent or absent. Much of the assistance they received was what others perceived they needed. Decision making and access to supports, controlled by others, affected identity and the capacity to function as independent adults. Experiences of emotional and physical abuse and manipulation by trusted individuals and institutions were prevalent. Without adequate resources and support, adults with DCC found it difficult to develop strategies for independence. They felt isolated and unheard. As a cohort, they expressed frustration that the management of their condition was fragmented and lacked accurate knowledge to guide it. Their capacity to grow and function effectively as independent adults had been compromised.

## Conclusion

As the first study to document the lived experience of a group of adults with corpus callosum disorders, this research begins to fill a knowledge gap. To live with a rare brain disorder without adequate social supports is highly significant. Participants felt excluded from key life domains and struggled with independence and identity as adults. To instigate truly effective change for this cohort, social research must tackle the issues of applicability and impact to alter the dominance of uninformed practices, hindered by prevailing myths that adults with a DCC must be unaffected if undiagnosed (O’Brien 1994, [40]).

This study identified systemic and knowledge gaps through first-hand experiences and perspectives shared by adults with a DCC. The participants described struggles to exercise control and fit into a world where they were expected to know how to function effectively but didn’t have all the skills, support or resources to do so. Although they identified areas of personal resilience and functional capacity, they described feeling at risk, barely coping and not having the strategies required to fulfil basic need*s*. Health professionals were perceived as lacking knowledge and experience to effectively deliver and manage the DCC diagnosis. Adults with a DCC perceived elements of society as misunderstanding and excluding them. To build relevant support systems, these perceptions require further exploration and understanding.

Although limited by the small sample size, findings of this study highlighted perceptions of barriers to educational and employment opportunities, affecting key outcomes for adults with DCCs. It identified perceived obstacles impeding access to mainstream and disability services. To more effectively navigate their lives, adults with a DCC would benefit from improved, coordinated supports based on informed practices that better recognised and understood their individual and group needs. Additionally, evidence that diagnosis specific support groups are valuable for people with rare conditions, substantiates exploration of developing a community to strengthen connection and self-advocacy [2, 34]. Clinicians, educators and allied health professionals would benefit from targeted prevocational training and access to evidence based, best-practice guidance and resources.

This study highlighted the urgent need for research to further explore the impacts of DCC on the lives of adults, in addition to understanding how professionals, families and the wider community can better comprehend their needs. Greater understanding and knowledge through lived experience and participatory research would provide a powerful instrument to inform best-practice guidelines. It would enable collaboration between researchers and the adults to identify and communicate their needs. It is of paramount importance for adults with DCCs to be involved and consulted at all stages of future research, enabling their needs to be identified and voices to be actively heard.

## Data Availability

Deidentified, transcribed interviews are stored on the University of Melbourne password protected, cloud storage platform, OneDrive. Only the named authors have access to this data.
